# *β*-Carboline dimers inhibit the tumor proliferation by the cell cycle arrest of sarcoma through intercalating to Cyclin-A2

**DOI:** 10.3389/fimmu.2022.922183

**Published:** 2022-10-17

**Authors:** Huiya Ma, Hongzhi Yu, Zhengyang Li, Zhi Cao, Youwei Du, Jiangkun Dai, Dongming Zhi, Yujie Xu, Na Li, Junru Wang

**Affiliations:** ^1^ College of Chemistry and Pharmacy, Northwest A&F University, Yangling, China; ^2^ College of Plant Protection, Northwest A&F University, Yangling, China; ^3^ Instrumental Analysis Center, Xi’an Jiaotong University, Xi’an, China

**Keywords:** β-Carboline-3-carboxylic acid dimers, CCNA2, CDK2, cell cycle, apoptosis, tumor-infiltrating cell

## Abstract

*β*-Carbolines are potentially strong alkaloids with a wide range of bioactivities, and their dimers exhibit stronger antitumor activity other than the monomers. However, the detailed mechanisms of the *β*-carboline dimers in inhibiting sarcoma (SARC) remain unclear. The results showed that *β*-carboline-3-carboxylic acid dimers Comp1 and Comp2, which were synthesized in our lab and modified at the N^9^ position and linked at the C^3^ position, exhibited effective inhibition activity on MG-63 proliferation (IC_50 =_ 4.6μM). Meanwhile, the large scale transcriptome profiles of SARC from The Cancer Genome Atlas (TCGA) were analyzed, and found that abnormal expression of genes relevant to apoptosis, cell cycle, and signaling pathways of Hedgehog, HIF, Ras involved in the SARC pathogenesis. Interestingly, both dimers could promote the apoptosis and arrest the cell cycle in S phase to inhibit proliferation of MG-63. Moreover, Comp1 and Comp2 inhibited the expression *CDK2*, *CCNA2*, *DBF4*, and *PLK1* associated with various immune cells and cell cycle in MG-63. Remarkably, drug-target interaction network analysis showed that numerous proteins involved in cell cycle were the potential targets of Comp1 and Comp2, especially CCNA2. Further molecular docking, isothermal titration calorimetry (ITC) and Cellular Thermal Shift Assay (CETSA) confirmed that both dimers could directly interact with CCNA2, which is significantly correlated with CD4+ T cells, by strong hydrophobic interactions (K_d_=5.821 ×10^6^ N). Meanwhile, the levels of CCNA2 and CDK2 were inhibited to decrease in MG-63 by both dimer treatments at transcription and protein levels, implying that Comp1 and Comp2 blocked the interaction between CCNA2 and CDK2 through competitive binding with CCNA2 to arrest the cell cycle of MG-63 cells in the S phase. Additionally, the transcriptome profiles of *β*-carboline-treated mice from Gene Expression Omnibus (GEO) were obtained, and found that similar antitumor mechanism was shared among *β*-carboline derivatives. Overall, our results elucidated the antitumor mechanisms of Comp1 and Comp2 through dual-suppressing the function of CCNA2 to profoundly arrest cell cycle of MG-63, then effectively inhibited cell proliferation of MG-63. These results provide new insights into the antitumor mechanism of *β*-carboline dimers and new routes of various novel cancer-related drug targets for future possible cancer therapy.

## Introduction

**Figure 1:**
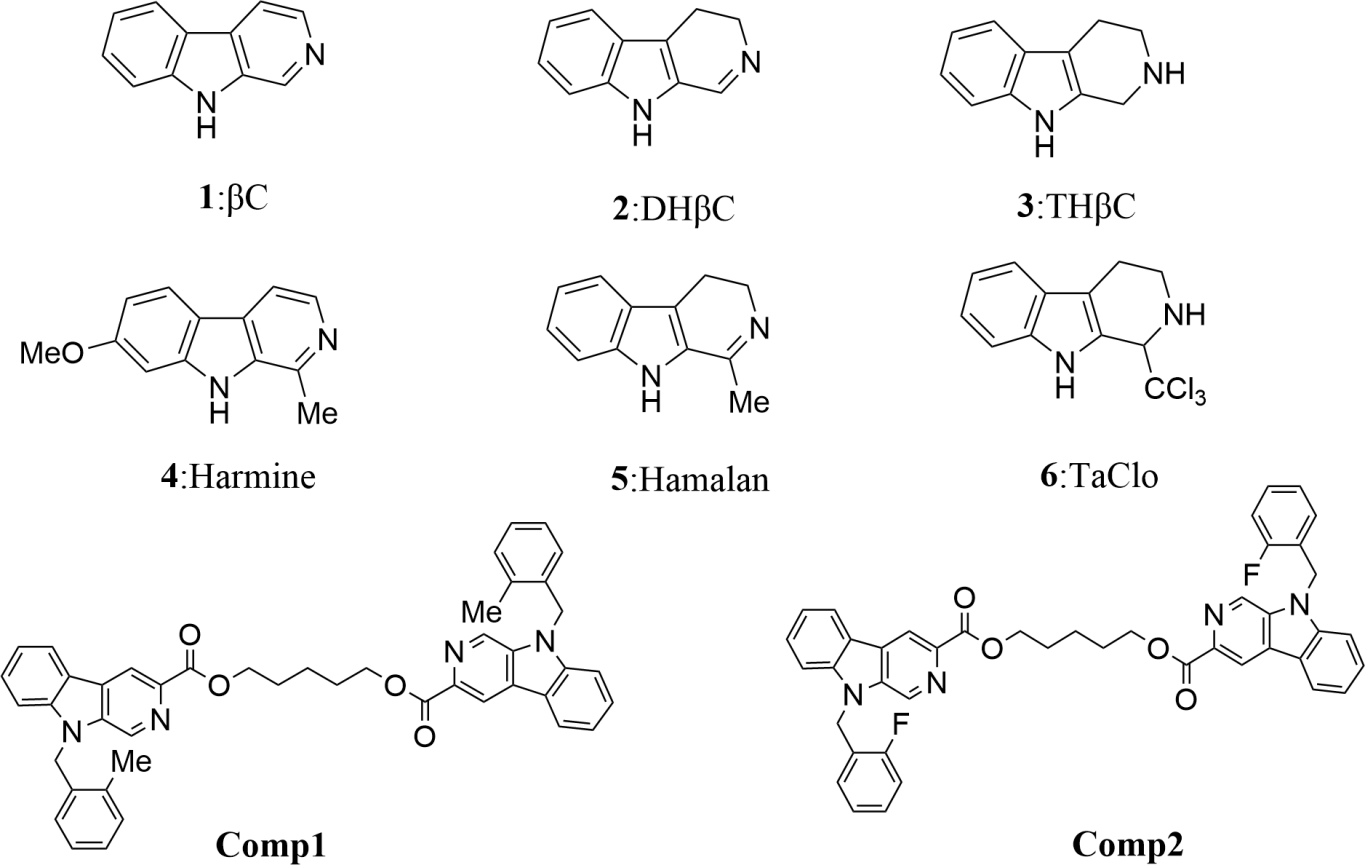
Structures of simple tricyclic *β-*carboline and *β-*Carboline-3-carboxylic acid dimers. Comp1:pentane-1,5-diyl-bis-(9-(2-methylbenzyl)-9H-pyrido[3,4-b]indole-3-carboxylate. Comp2:pentane-1,5-diyl-bis-(9-(2-fluorobenzyl)-9H-pyrido[3,4-b]indole-3-carboxylate.

*β-*carboline alkaloids have a unique core structure with the tricyclic pyrido-[3,4-b] indole ring (**Figure 1**) (1) and exhibit outstanding bioactivity, including antitumor, antitubercular, antimalarial, sedative, hypnotic, anticonvulsant, antimicrobial, antiviral, etc (2). And their antitumor activities generate the greatest interest in the pharmacological area (3). In the last decades, several types of tumors including prostate, pancreatic, breast, lung, and sarcoma cancers, are proven to be significantly inhibited by *β-*carbolines, especially in dimer forms. In recent years, mechanistic studies show that *β*-carboline monomers exert antitumor activities mainly by intercalating into DNA base pairs (4), and inhibiting topoisomerase I and II (5), CDK (6), MAPKAP-K2 (7), MK-2 (8), I*k*B kinase and kinesin (9). Meanwhile, *β*-carboline also influences tumor progression by affecting representative proteins involved in cell cycle, apoptosis, autophagic, metastasis, tumor immunity, etc (10). However, the bioactivity of *β-*carboline dimers in the treatment of sarcoma and the underlying mechanism is still unknown.

Of note, the sarcomas (SARCs) represent an extensive group of malignant diseases derived from mesenchymal cells (11). Statistically, more than 15,000 diagnoses and deaths annually in the US are associated with SARC (12). Compared with the low incidence in adults, the SARCs account for a higher percentage of overall cancer morbidity and mortality in children and young adults (ages 20–39) (13). Chemotherapy has also been proven helpful in achieving remission of SARCs, in which alkaloid compounds, like oxymatrine, piplartine, and piperine, show effective anti-sarcoma activities (14, 15). Remarkably, numerous *β-*carboline derivatives exhibit active antitumor activities, such as 6-,8- or 6,8-substituted 3-benzylamino-*β-*carboline derivatives with IC_50_<1.00 μM against Sarcoma 180 cells (16). *β-*carboline linked arylsulfonyl piperazine derivatives also performed strong inhibition of cell growth in the human osteosarcoma cell line (17). Thus, elucidating the detailed mechanisms of anti-tumor activity of *β-*carboline derivatives will contribute to developing and using novel anti-tumor drugs in clinic.

The results of the present study showed that the *β*-carboline-3-carboxylic acid dimers, synthesized in our lab, performed effective inhibition activity on MG-63 cells. Then, the clinical multi-transcriptome profiles, network pharmacology and molecular measures performed in-depth analysis to elucidate the underlying anti-tumor mechanisms of Comp1 and Comp2. The results showed that *β*-Carboline-3-carboxylic acid dimers Comp1 and Comp2 could arrest cell cycle at S phase and promote apoptosis in MG-63 to inhibit proliferation of MG-63. Importantly, Comp1 and Comp2 are directly inserted into Cyclin-A2 (CCNA2) to block CCNA2-CDK2 interaction, leading to an effective arrest of cell cycle at S phase in SARC. Meanwhile, the results identified that *β*-carboline-related derivatives exhibited similar anti-tumor mechanisms that *β*-carbolines mainly inhibited expressions of genes involved in tumor immunity, pathogenesis of SARC, and especially cell cycle to exert their antitumor activities. Overall, these results deepened our understanding of the antitumor mechanisms of carboline, thus providing a novel strategy for the development of new carboline-related antitumor drugs and new drug targets for accurate cancer treatment.

## Materials and methods

### GEO and TCGA data analysis and DEGs identification

The gene expression profiles of GSE150563 (GSM4551578-GSM4551583) were downloaded from the Gene Expression Omnibus (GEO) database (https://www.ncbi.nlm.nih.gov/), and gene expression data of harmine untreated WT mice (WTU) and three harmine treated WT mice (WTT) were obtained (18). The sequencing organism samples are *Mus musculus*. Principal component analysis (PCA) was performed on the gene expression profiles obtained from GEO to reduce the dimensionality and evaluate the independence of WTT and WTU samples. The DEseqR package was used to identify the differentially expressed genes (DEGs) in GEO datasets in the R platform (Version 3.7.1). The threshold for DEG is *P* < 0.05 and Fold change > 2.

The Cancer Genome Atlas (TCGA), a project supported by the National Cancer Institute (NCI) and National Human Genome Research Institute (NHGRI), has generated comprehensive and multidimensional maps of the key genomic changes in various types of cancers. DEGs were then identified using the DEseqR package of R statistical software (fold change > 2 and *P* < 0.05 were set as the cut-off values). Subsequently, the differentially expressed genes acquired from the GEO and TCGA databases were combined to obtain the hub genes. The Immune infiltration analysis was performed on related genes and six types of tumor infiltrating immune cells (TIICs), including CD4+ T cells, CD8+ T cells, B cells, neutrophils, and dendritic cells (DCs), and macrophages using the “Immune-Gene” module of TIMER2 web server based on TIMER algorithms (19). The purity of tumors was quantified, then Ps and partial correlation values (cor) were calculated through the purity-adjusted Spearman’s rank correlation test.

### Functional enrichment analysis and reconstruction of the protein-protein interaction network 

The Kyoto Encyclopedia of Genes and Genomes (KEGG) pathway and GO annotations (GO) enrichment analyses of DEGs were performed in the R package cluster profile software based on the Benjamin-Hochberg method (*P* < 0.05). Protein-protein interaction network data of candidate DEGs were constructed using the Search Tool for the Retrieval of Interacting Genes (STRING) database (http://string-db.org) (20). Cytoscape software (version 3.7.0; https://cytoscape.org/) was used to visualize the interaction networks of the candidate DEGs. Gene set enrichment analysis (GSEA) for pathway enrichment results was performed by the GSEA version 4.0 portal (21). Enrichment analysis was performed and showed a change from a high enrichment score to a low enrichment score, with a threshold of *P* < 0.05.

### A network pharmacology approach and molecular docking study

Public PharmMapper Server databases (http://lilab.ecust.edu.cn/pharmapper/) were employed to identify compound-related targets depending on chemical similarities and pharmacophore models (22). The two *β-*carboline compounds (**Figure 1**) that were synthesized in our laboratory were used to predict the target. Based on the reverse pharmacophore matching method, the pharmacophore model of the drug was selected, and the final 300 protein conformations were set to obtain the target name, gene name, Universal Protein (UniProtID), fit score, and other results related to the compound.

The docking programs Autodock 4.2 and Autodock Vina were used to dock DBF4 and CCNA2 (PDB ID: DBF4: 4F99; CCNA2: 1E9H) to test the activity of the selected compounds. The sum of hydrogen bonds and hydrophobic interactions was used to compute the free energy of binding in semiflexible docking simulations. In Autodock with default values, the total of the lowest predicted free energy from possible binding conformations of each ligand was determined and ordered by binding free energies.

### *β*-Carboline samples, cell culture, and the proliferation assay

The synthesis of *β-*carboline-3-carboxylic acid dimers, as described in a prior article (23). MG-63 and HL7702 cells were grown in Gibco’s modified Eagle’s medium (DMEM) supplemented with 10% fetal bovine serum (FBS, HyClone), 100 IU/ml penicillin, and 100 µg/ml streptomycin (Gibco, 12100-061). All cells were bought from RuYao Biotechnology Company and kept at 37°C in a humidified atmosphere of 5% CO_2_ (Zhejiang, China).

The cells (5x10^4^ cells/ml) were seeded in 96-well plates (five wells per treatment group) and treated with various doses of Comp1, Comp2, and doxorubicin (DOX) at 37°C. Cell viability was determined using methyl thiazol tetrazolium (MTT) (Sigma, USA) after a 48-hour incubation period by measuring optical density at a wavelength of 490 nm using a microplate reader (Denmark; BioRad).

### Reverse transcription and quantitative real-time polymerase chain reaction

TRIzol reagents were used to extract total RNA (Life Technologies, USA). Total RNA cDNA synthesis was carried out according to the manufacturer’s instructions using an EasyScript cDNA Synthesis Kit (Transgene, China). The 2^−ΔΔCt^ technique was used to measure the expression levels of *CCNA2, DBF4, PLK1, CDK2, MMP7, TK1*, and 15 additional genes (**Table S1**). The experiment was at least three times repeated with three technical replicates.

### Flow cytometry and staining assay

The MG-63 cells (2x10^5^/ml) in 2 ml were plated in 6-well plates and treated with Comp1 and Comp2 at 2.5 μM, 5 μM, and 10 μM concentrations for the test. For 48 hours, treated cells were cultured at 37°C in a 5% CO_2_ incubator. Cell apoptosis was detected by Annexin V-fluorescein isothiocyanate (FITC)/propidium iodide (PI) double labeling. The cell cycle was detected by 20 µL PI (50 µg/mL) (Sigma Aldrich, Saint Louis, MO, USA) for 30 min at 37°C. The samples were analyzed using flow cytometry (BD FACSCalibur, San Jose, CA, USA).

Hoechst 33342 and PI dual staining performed the morphological observation of apoptosis according to the manufacturer’s instructions. MG-63 cells (2x10^4^ cells per well) seeded into 24-well plates were incubated with DMEM containing 5 µM of Comp1 and Comp2 for 48 h at 37 °C and 5% CO_2_. Then, the cells were stained by Hoechst 33342 and PI at 37 °C for 30 min in dark conditions. The cells cultured as described above were stained with DAPI at 37°C for 15 min (10 μg/mL in PBS) in darkness. After being washed with PBS, cells were observed and photographed with a fluorescence microscope.

### Cellular thermal shift assay and Western blot analysis

CETSA was performed as previously reported, with some modifications (24). Briefly, MG-63 cells were incubated with DMEM at 37°C and 5% CO_2_. Cells were washed with ice-cold PBS, lysed with ice-cold M-PER reagent, and supplemented with protease and phosphatase inhibitor cocktails on ice for 10 min. The supernatants were incubated with DMSO or the corresponding compound (200 μM) at 4°C overnight. After incubation, the samples were divided into 100 μL aliquots and heated at the indicated temperature for 10 min in a PCR machine, followed by 10 min of cooling on ice. The soluble proteins were separated by centrifugation and subjected to Western blot analysis.

Western blotting Cells were plated in 6-well plates and incubated with compounds at different concentrations for the indicated time. Cells were then collected and detected by standard Western blot as described before (24). The intensities of target bands were analyzed using the Image J software 1.52 v (NIH, USA).

### Protein expression and purification

The human *CCNA2* gene’s coding sequence was cloned into the GST-tagged PGEX-4T vector for protein production and purification (Transheep Biotechnology, Shanghai, China). After that, the recombinant 4T was converted into BL21 (DE3) to express *CCNA2*. Positive clones were tested for in-frame insertion using PCR and sequenced. After the bacterial culture had grown to an optical density (OD_600_) of around 0.6 at 600 nm, isopropyl-D-thiogalactopyranoside (IPTG) was added to promote the production of the CCNA2-GST protein for 4 hours at 37°C. The recombinant CCNA2 protein (known as rCCNA2) was purified according to the manufacturer’s procedure using a glutathione S-transferase (GST) tag protein purification kit (Beyotime Biotechnology, Shanghai, China). Enterokinase digestion cleaved the upstream 43-residue N-terminal fusion sequence from purified fusion proteins. The proteins that flowed through were collected and dialyzed against TBS glycerol buffer. The BCA technique was used to determine the concentration of pure rCCNA2.

### Co-Immunoprecipitation

For Co-IP assays, recombinant plasmid of CDK2 and CCNA2 was fused with GFP and MYC tag for acquiring CDK2-GFP and CCNA2-MYC proteins, respectively. Then, HEK-293 T cells were co-transfected with the plasmids pc-CDK2:GFP and pc-CCNA2:MYC. At 48 h post transfection (hpt), the cells were lysed and the supernatant was co-incubated with equilibrated Anti-GFP magnetic beads (Thermo Scientific, San Jose, CA, USA) or anti-GFP magnetic beads and comp1 or comp2 (10 μM), and the subsequent steps were performed following Kun et al. (2022, 25).

### Isothermal titration calorimetry

On an iTC200, ITC tests were performed (Nano ITC). As a ligand, the CCNA2 protein solution (9 mM in PBS, 50 L) was titrated into Comp1 and Comp2 (0.05 mM) in PBS with a volume of 2.5 μL for each titration, and water was used as a control.

## Results

### Harmine altered the transcription pattern of genes relevant to cancer pathogenesis in mice

**Figure 2:**
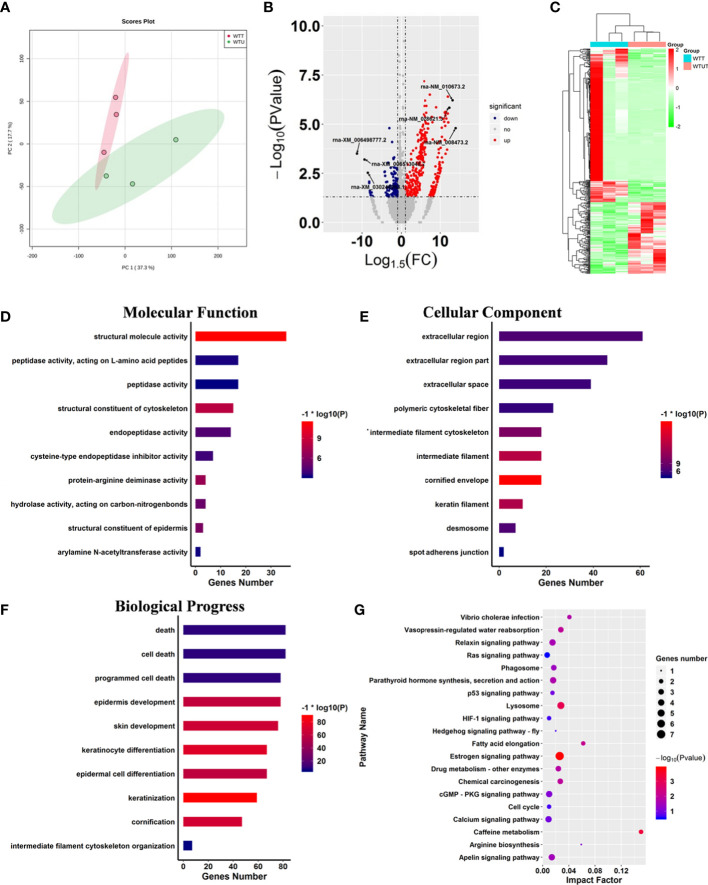
Harmine affected cancer-related progress. **(A)** Principle component analysis displaying the observable variation in the gene expression pattern under harmine treatment. The points in the red region represent the harmine-treated samples, whereas the points in the green region represent the control samples. **(B)** Volcano plot of the differentially expressed genes in response to harmine treatment. The down- and upregulated genes are shown in blue and red, respectively (log2fold change < -1 or > 1; *P* < 0.05). **(C)** Heatmap showing two different gene expression patterns under harmine treatment. The up-and downregulated genes are shown in red and green. Each column represented a sample in the GEO dataset. **(D-F)** Gene ontology enrichment analysis of the differentially expressed genes; **(D)** molecular function; **(E)** cellular component; **(F)** biological progress; **(G)** Kyoto Encyclopedia of Genes and Genomes pathway analysis of the differentially expressed genes. The size of the circle indicates the number of genes involved in the pathway, whereas the circle color represents the significance of enriched pathways (*P* < 0.05).

Harmine is a typical carboline, and contains a hub carboline ring which is responsible for the activities of carboline derivatives. To investigate the influence of carboline on the gene expression pattern of mice, a set of GEO data of harmine was harvested and analyzed. Two algorithms including PCA and correlation analysis were used to distinguish the differences between WTT and WTU treatment from GEO data (**Figure 2A**). Both algorithms reached a consensus that all samples from the same treatment were closely clustered into one region, and eventually formed two groups represented by WTT and WTU, validating harmine experimental treatment (**Figure 2A**). These results suggested that harmine significantly altered the gene expression pattern in mouse skeletal muscle. In the WTT vs. WTU comparison, 452 genes that matched the threshold (log2fold change < -1 or > 1, *P* < 0.05) were recognized as differentially expressed genes (DEGs, **Figure 2B**). Among all DEGs, 312 genes were upregulated in WTT, while 140 genes were downregulated in WTU (**Figure 2B**). As shown in Fig. 2C, the heatmap of gene expression observably showed that genes with similar expression patterns stably clustered together according to experimental treatment, suggesting their close association with the effect of harmine (**Figure 2C**).

To investigate the influence of harmine on the biological processes of mice, KOG and GO analyses were performed on all DEGs (**Figure 2**). The results of the Clusters of Orthologous Groups of proteins (KOG) analysis indicated that harmine mainly influenced the genes that functioned in transcription, signal transduction mechanisms, cytoskeleton, posttranslational modification, protein turnover, chaperones, and extracellular structure (**Figure S1A**). Further GO enrichment analysis showed that ten categories closely correlated with molecular function were significantly enriched, including structural molecule activity, structural constituent of the cytoskeleton, protein-arginine deiminase activity, structural constituent of the epidermis, and hydrolase activity (**Figure 2D**). In parallel, the overrepresentation of various terms relevant to cellular components was identified, such as cornified envelope, intermediate filament, and keratin filament (**Figure 2E**). Remarkably, fifty-nine terms relevant to biological progress were significantly influenced by harmine treatment, such as programmed cell death, cell death, intermediate filament cytoskeleton organization, keratinization, and keratinocyte differentiation (**Figure 2F**). Moreover, various significant terms above are closely correlated with cancer-related immunity and progress (26). Therefore, these results implied that *β-*carboline could affect the expression of genes involved in cancer pathogenesis.

Subsequently, pathway analysis was performed on DEGs against the KEGG database. The results showed that 23 biosynthesis pathways were significantly enriched under harmine treatment, such as the hedgehog signaling pathway, cell cycle, HIF-1 signaling pathway, Ras signaling pathway, lysosome, and phagosome (**Figure 2G**). To further identify the most active pathways associated with harmine treatment, Gene Set Enrichment Analysis (GSEA) was performed on the pathway enrichment results (**Table 1**). The results showed that the most active pathway was PPAR signaling pathway which positively correlated with harmine treatment, with the highest normalized enrichment score (NES) of 1.34 (**Table 1**). In contrast, five pathways exhibited significantly negative correlation with harmine treatment, including the Ras signaling pathway, lysosome, cell cycle, MAPK signaling pathway, and apelin signaling pathway (**Table 1**). Among these five pathways, the Ras signaling pathway was the most active process *in vivo* following harmine treatment, with the lowest NES of -1.22 (**Table 1**). As the downstream process of the Ras signaling pathway, the cell cycle was also dramatically suppressed by harmine, with an NES of -1.11, indicating the negative effect of harmine on expression of genes relevant to cell cycle. Remarkably, most of these pathways were involved in cancer immunity and pathogenesis (27, 28). Taken together, the results showed that *β-*carboline represented by harmine will alter expression patterns of genes involved in numerous biological processes relevant to the pathogenesis of cancer, especially cell cycle, Ras and PPAR signaling pathways.

**Table 1:** The results of the enrichment score of each pathway by GSEA.

NAME	SIZE	NES
Ras signaling pathway	2	-1.21695
Lysosome	4	-1.18953
Cell cycle	1	-1.10642
MAPK signaling pathway	1	-1.01016
Apelin signaling pathway	3	-1.00313
Calcium signaling pathway	3	-0.95861
Phagosome	2	-0.95375
Pathways in cancer	2	-0.94977
HIF-1 signaling pathway	1	-0.92393
P53 signaling pathway	1	-0.92129
PI3K-AKT signaling pathway	2	-0.72041
CGMP - pkg signaling pathway	3	-0.58098
AMPK signaling pathway	1	1.072363
CAMP signaling pathway	2	0.498206
Drug metabolism - other enzymes	2	0.654187
PPAR signaling pathway	1	1.335199

### Changes in expression of genes relevant to cell cycle were the hub variations involved in SARC pathogenesis

**Figure 3:**
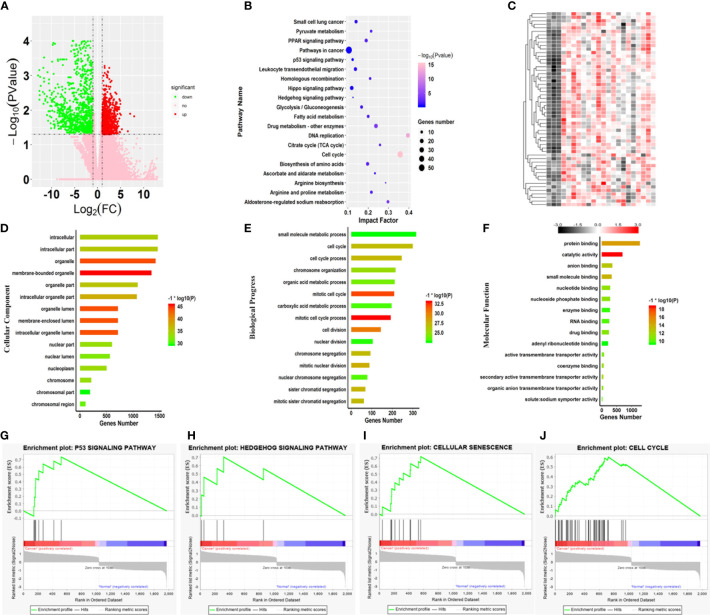
Core variations between SARC and normal tissue. **(A)** Volcano plot of the aberrant genes of SARC and noncancer samples from TCGA. **(B)** Kyoto Encyclopedia of Genes and Genomes pathway analysis of the differentially expressed genes. **(C)** Heatmap of genes differentially expressed genes involved in key pathways in the SARC dataset. Gene ontology enrichment analysis of the differentially expressed genes; **(D)** Cellular component; **(E)** Biological process; and **(F)** Molecular function. **(G–J)** Enrichment plots from gene set enrichment analysis (GSEA); **(G)** P53 signaling pathway, **(H)** Hedgehog signaling pathway, **(I)** cellular senescence, **(J)** cell cycle.

To identify the hub genes associated with SARC, 263 transcriptome profiles of pathological tissue from SARC patients and two normal tissues were collected from TCGA for further analysis. Then, the transcriptome profiles were analyzed by edgeR software based on the R platform to identify DEGs associated with the SARC pathogenesis. In total, 1977 genes were identified as DEGs, with 1037 upregulated and 940 downregulated genes in SARC (**Figure 3A**).

Subsequently, GO enrichment analysis was performed on all 1977 DEGs. For cellular components, 182 terms were significantly overrepresented in SARC, and these genes mainly functioned in membrane-bound organelles, organelles, membrane-enclosed lumens, organelle lumens, intracellular organelle lumens, intracellular organelle parts, and organelle parts (**Figure 3D**). In terms of molecular function, 138 categories were significantly enriched against these DEGs, including catalytic activity, protein binding, small molecule binding, anion binding, secondary active transmembrane transporter activity, coenzyme binding, nucleotide-binding, and nucleoside phosphate binding (**Figure 3E**). Importantly, it was also found that the changes in expression of genes involved in cell cycle that 769 terms of biological progress including cell cycle process, cell division, mitotic nuclear division and sister chromatid segregation were significantly altered in SARC tissue (**Figure 3F**). Of note, these terms were cancer-related gene ontologies (29, 30), and were also affected by harmine treatment.

The progress of cancerous turning is always accompanied by changes in metabolic pathways (31, 32). Thus, pathway enrichment analysis against the KEGG database was further performed on the DEGs associated with SARC (**Figure 3B**). The results showed significant changes in 63 pathways in SARC, including cell cycle, aldosterone-regulated sodium reabsorption, PPAR signaling pathway, arginine biosynthesis, DNA replication, valine, leucine, glycolysis/gluconeogenesis, and isoleucine degradation and spliceosome (**Figure 3B**). Remarkably, the most significant pathway was the cell cycle, with the lowest P value (**Figure 3B**). Changes in the expression of genes involved in the cell cycle are recognized as the key variation in cancer immunity and progression (33, 34). Thus, the abnormal expression of genes responsible for cell cycle involved in the SARC pathogenesis was highlighted.

To identify pathways that were dramatically activated in SARC, the GSEA on TCGA data (**Figure 3**) was conducted. GSEA results showed that 4 pathways were most significantly enriched and positively correlated with the pathogenesis of SARC, including cellular senescence, p53 signaling pathway, cell cycle, and hedgehog signaling pathway (**Figures 3G–J**). The transcription of all genes involved in these pathways was dramatically upregulated in SARC tissue (**Figure 3C**). And the most active pathway was cellular senescence, with the highest NES of 1.39 (**Table 1**). Moreover, cellular senescence, P53 signaling pathway, cell cycle, and hedgehog signaling pathway are involved in tumor immunity in various cancer types (35). The results observed that these four pathways were also overrepresented in the GEO data, implying that *β-*carboline could potentially affect the expression of genes relevant to the pathogenesis and immunity of SARC through influencing cellular senescence, cell cycle, p53 and hedgehog signaling pathways (**Figure 3**). Therefore, the genes that functioned in these pathways were recognized as the focus to further elucidate the anti-tumor mechanisms of our carboline derivatives.

### Genes relevant to the cell cycle and immunity of SARC were potential targets of *β*-carboline

**Figure 4:**
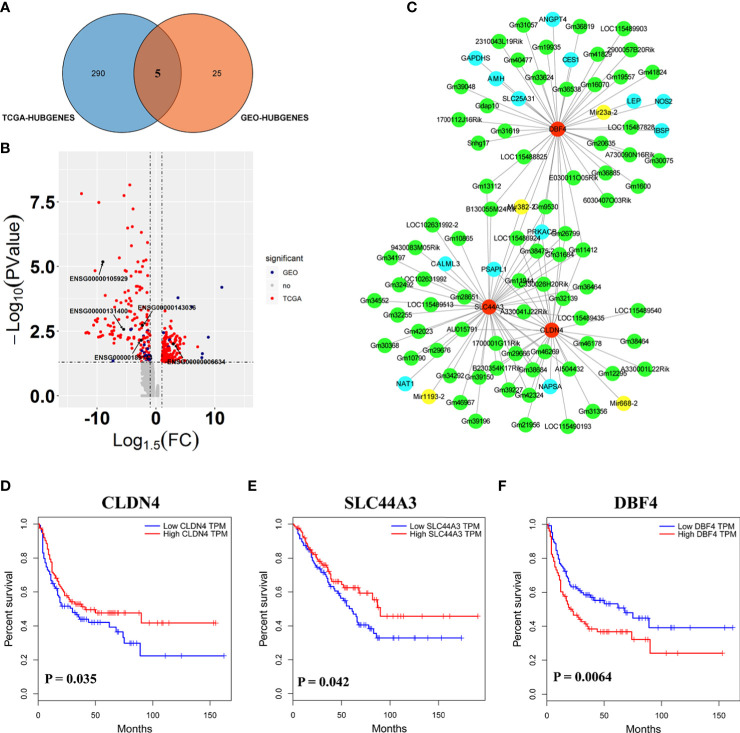
Three genes played important roles in the potential mechanism of harmine-treated cancer. **(A)** The Venn diagram demonstrates the intersections of genes between GEO data and TCGA data. **(B)** Volcano plot of genes functioned in key pathways identified in TCGA and GEO datasets. **(C)** The co-expression network of ceRNAs. Nodes and edges represent the proteins and interaction relationships between DEGs, respectively. Red nodes indicate the key genes discussed in the present manuscript, green nodes indicate lncRNAs, blue nodes indicate hub genes, and yellow nodes indicate miRNAs. **(D–F)** Disease-free survival (RFS) analysis for GEO and TCGA consensus genes, P <0.05 (*SLC44A3*, *DBF4*, and *CLDN4)*. The blue line represents SARC patients with relatively low *SLC44A3*, *DBF4*, and *CLDN4* mRNA expression, and the red line represents SARC patients with high expression of the aforementioned genes.

Here, the 295 and 30 hub genes that functioned in candidate pathways from TCGA and GEO data profiles were identified, respectively (**Figure 4A**). By the gene expression value of TCGA, 158 hub genes were upregulated, whereas 137 hub genes were downregulated in cancer tissue (**Figure 4B**). Among the 30 hub genes from GEO, there were 20 downregulated genes and 10 upregulated genes. Interestingly, the results observed that 5 hub genes were shared in the GEO and TCGA profiles, including *SLC44A3*, *DBF4*, *NAPSA*, *ATP6V0A4*, and *CLDN4*, which were closely associated with B cells, CD4+ T cells, CD8+ T cells, Macrophage, Neutrophil, and Dendritic cells to involve in tumor immunity (**Figure 4A**; **Figures S5, S6**). By the GEO data, *SLC44A3*, *DBF4*, *NAPSA*, and *ATP6V0A4* were all downregulated under harmine treatment, whereas *CLDN4* was induced to upregulate by harmine (**Figure 4B**).

To further evaluate the importance of these 5 genes in sarcoma, Kaplan–Meier analysis was performed against clinical data (**Figures 4D–F**). Kaplan–Meier survival analysis showed that SARC with *CLDN4*-low had a worse prognosis than that with *CLDN4*-high (*P*=0.035). Furthermore, *CLDN4* was upregulated in SARC, and *CLDN4* was downregulated in GEO (**Figures S2A–C**). The results showed that a low level of *SLC44A3* was significantly (*P*=0.042) associated with a shorter period of sarcoma tumor patients, and patients with a low expression level of *SLC44A3* were more serious than those with a high expression group. However, the expression of this gene was downregulated in both datasets. Here, a high level of *DBF4* associated with lower survival was noted.

Additionally, a complex regulatory network involved in the effect of harmine on tumors was identified. The expression of *SLC44A3*, *DBF4*, and *CLDN4* closely correlated with 76 lncRNAs, including B230354K17Rik, C330026H20Rik, E030011O05Rik, Gdap10, Gm10790, Gm10865 and Gm11412, and 4 miRNAs, including Mir1193-2, Mir23a-2, Mir382-2 and Mir668-2 (**Figure 4C**, **Figure S1B**). It has been well documented that miRNAs and lncRNAs play essential roles in regulating genes involved in various biological processes associated with tumor pathogenesis (36). Thus, the results proposed that *β-*carboline complexly reprogrammed SARC-related biological processes by affecting a series of transcripts represented by functional genes, lncRNAs, and miRNAs.

### *β-*Carboline-3-carboxylic acid dimers inhibit SARC through arresting cell cycle and promoting apoptosis in MG-63

**Figure 5:**
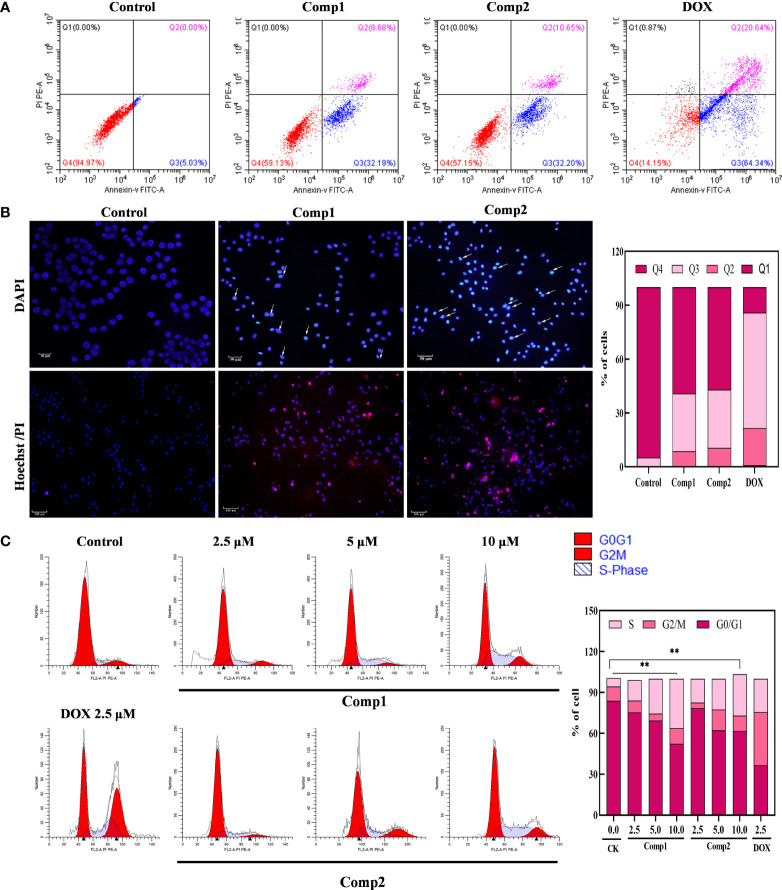
*β*-Carboline-3-carboxylic acid dimers promoted the apoptosis and induced arrest of cell cycle at S phase in MG-63 cells. **(A)** *β-*Carboline-3-carboxylic acid dimers induce apoptosis in MG-63. MG-63 cells were treated with Comp1 and Comp2 (5 μM) for 48 h, and cells were then stained with Annexin V-fluorescein isothiocyanate (FITC)/propidium iodide (PI) double labeling and subjected to flow cytometry. **(B)** Morphological observation was performed by Hoechst 33342/propidium iodide (PI) dual staining and DAPI staining. MG-63 cells seeded into 24-well plates were incubated with DMEM containing 5 µM of Comp1 and Comp2 for 48 h. **(C)** *β-*Carboline-3-carboxylic acid dimers induce cell cycle arrest. MG-63 cells were treated with Comp1 and Comp2 (2.5 μM, 5 μM, 10 μM) for 48 h, and the cell cycle distribution was analyzed by flow cytometry. The percentages of cells in the G1, S, and G2/M phases are presented statistically. **P < 0.01.

The antiproliferative effect of *β-*carboline-3-carboxylic acid dimers (**Figure 1**) on MG-63 cells was evaluated *via* MTT assay. Comp1 and Comp2 displayed observable inhibitory activities against MG-63, with IC_50_ values of 4.607 μM and 4.972 μM, respectively (**Table 2**). The inhibitory activity of Comp1 and Comp2 on control HL7702 cells was significantly lower than that on MG-63, with IC_50_ values of 10.083 μM and 9.525 μM, respectively, demonstrating the advantages of *β-*carboline-3-carboxylic acid dimers in antitumor activity. Considering the function of carboline-like compounds in triggering cell death and apoptosis in tumor cells, the results proposed that our Comp1 and Comp2 may also cause apoptosis of MG-63. Thus, DAPI staining, Hoechst 33342/propidium iodide (PI) dual staining assay, and Annexin V/PI double staining were performed in MG-63 cells that were treated with *β-*carboline-3-carboxylic acid dimers. The number of the MG-63 cells markedly decreased (**Figure 5A**) and the cell condition gradually deteriorated following Comp1 and Comp2 treatments. The apoptosis rate of MG-63 cells was increased in the Comp1 and Comp2 groups (*P*<0.05), as shown by flow cytometric evaluation of the Annexin V/PI double-stained cells (**Figure 5A**). Further DAPI staining showed that the combination of Comp1 and Comp2 induced nuclear chromatin condensation and granular apoptotic bodies (**Figure 5B**). To further identify the typical features of apoptosis, Hoechst 33342/propidium iodide (PI) dual staining assay was performed. The results showed that MG-63 cells in the control group were evenly dyed weak blue with a clear edge. However, after incubation with Comp1 and Comp2 (5 µM) for 48 h, the cells were stained with strong blue fluorescence and strong red fluorescence, and shrinkage, and condensation or fragmentation was observed (**Figure 5B**, **Figure S3**). These data suggested that *β-*carboline-3-carboxylic acid dimers exert their inhibitory effect on SARC cell proliferation by inducing apoptosis.

**Table 2:** *In vitro* anticancer activity (IC_50_ in μM) of *β-*Carboline-3-carboxylic acid dimers.

Cell line	DOX^a^	Comp1	Comp2
MG-63	1.122 ± 0.490	4.972 ± 0.863	4.608 ± 0.290
HL7702 (Control)	2.403 ± 0.172	10.083 ± 1.273	9.525 ± 0.377

a Doxorubicin used as a positive control.

The cell cycle has been documented in tumor immunity and apoptosis in various cancer types (37). Considering the effect of carboline in inhibiting genes relevant to cell cycle associated with SARC, the effect of different concentrations of *β*-carboline-3-carboxylic acid dimers on cell cycle distribution was tested. As shown in **Figure 5C**, when the concentration of Comp1 and Comp2 was 10 µM, the percentages of cells in the S phase increased to 36.23% and 30.46% (control group 6.17%) respectively, indicating that Comp1 and Comp2 could induce cell cycle arrest at the S phase in MG-63 cells. Overall, these results suggested that our dimers inhibit SARC by promoting apoptosis and arresting the cell cycle in S phase in MG-63.

### *β-*Carboline-3-carboxylic acid dimers inhibit SARC mainly by affecting the genes involved in the cell cycle and tumor immunity

**Figure 6:**
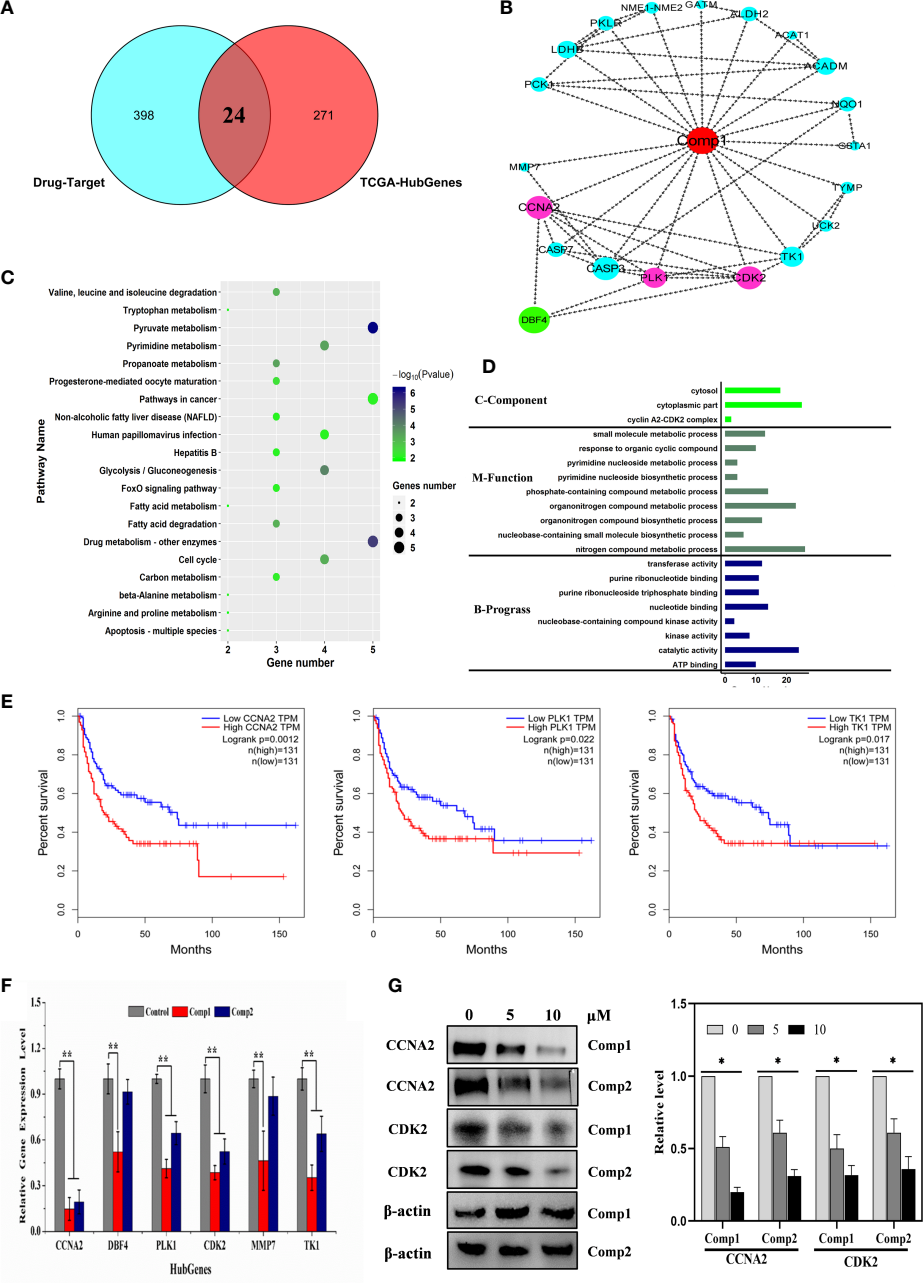
Cell cycle-related factors associated with cancer were affected by carboline. **(A)** The Venn diagram displays the common genes shared by the SARC and predicted drug-target profiles. **(B)** PPI network analysis of 19 hub genes. The network was constructed using the CytoHubba plugin in Cytoscape, and the core gene scores were calculated using the maximal clique centrality (MCC) method. The deeper the color of the node is, the higher the PPI score of the targets, and the red nodes represent the *β-*carboline-3-carboxylic acid dimers. **(C)** KEGG pathway analysis of 24 targets from Venn analysis. **(D)** Differential and functional enrichment analysis of 24 targets shared by SARC-related transcriptomic profiles and drug-target networks. **(E)** Disease-free survival analysis for predicted target genes and TCGA consensus genes, P <0.05 (*CCNA2*, *PLK1*, and *TK1*). **(F)** mRNA expression of *CCNA2*, *DBF4*, *TK1*, *PLK1*, *MMP7*, and *CDK2* in MG-63 cells detected by RT–qPCR. **(G)** Western blot analysis of CCNA2 and CDK2. MG-63 cells were treated with Comp1 and Comp2 at 5 and 10 µM for 48 h. ACTB was used as an internal control. *P < 0.05, **P < 0.01.

To further interpret the influence of *β-*carboline-3-carboxylic acid dimers, synthesized by our lab, on cancer-related biological processes, an effective drug-target protein interaction network of Comp1 and Comp2 was constructed using network pharmacology methods. Both compounds were predicted to have a total of 452 potential targets. In total, 398 targets were further identified based on the norm fit >0.4. Meanwhile, the results observed 24 targets shared by SARC-related transcriptomic profiles and the drug-target network (**Figure 6A**). And nineteen of 24 targets were identified in a complex interaction network (**Figure 6B**). KEGG and GO enrichment analysis showed that these 19 drug-targeted proteins interacting with *β-*carboline-3-carboxylic acid dimers mainly functioned in cancer-related pathways, including pyrimidine nucleoside metabolic processes, glycolysis/gluconeogenesis, and especially apoptosis and cell cycle (**Figures 6C, D**). Furthermore, the results showed that our *β-*carboline-3-carboxylic acid dimers may directly influence *CASP7*, *CCNA2*, *ERBB4*, *MMP7*, *PLK1*, and *TK1*. These six genes are involved in tumor immunity of SARC through correlating with B cell, CD4+ T cell, CD8+ T cell, Macrophage, Neutrophil, and Dendritic cells of SARC (**Figures S5, S6**). To explore the potential roles of individual targets in disease-free survival (**Figure 6E**), Kaplan–Meier survival curves from the GEPIA database were generated (35). Survival analysis indicated that patients with overexpression of these six targets exhibited significantly shorter survival times than patients with relatively low expression levels of representative genes (*P*<0.05), supporting the close connection of these six proteins with the prognosis of SARC. Importantly, the *β-*carboline-3-carboxylic acid dimer treatment dramatically suppressed the expression of *CCNA2*, *DBF4*, *TK1*, *PLK1*, *MMP7*, and *CDK2* to downregulate in MG-63 cells (**Figure 6F**).

Taken together, these results suggested that *β-*carboline-3-carboxylic acid dimers could arrest the cell cycle by suppressing *TK1*, *DBF4*, *CCNA2*, *CDK2*, and *PLK1*, together with influencing some cancer-related pathways to inhibit MG-63. Additionally, the inhibitory effect of harmine on the expression of *DBF4*, *CDK2* and *PLK1*, supported our speculation that similar antitumor mechanisms were shared by carboline derivatives.

### *β-*Carboline-3-carboxylic acid dimers arrest cell cycle of MG-63 through interacting with CCNA2 and blocking CCNA2-CDK2 interaction

Considering the imperative function of CCNA2 and CDK2 at the S/G2 phase in MG-63 cells, the results proposed that CCNA2 and CDK2 were the potential key targets in the anti-SARC mechanisms of *β*-carboline-3-carboxylic acid dimers. Based on the EPIC algorithm, *CCNA2* and CDK2 expression levels in SARC were negatively correlated with the infiltration level of CD4+ T cells (cor = −0.194, P = 2.62e−03; **Figures S6, S5**), implying that our dimers also could target CCNA2 and CDK2 to affect the tumor immunity in MG-63 cells. Indeed, our RT-qPCR assay has shown the suppression of dimers on the expression of *CCNA2* and *CDK2* in MG-63. Thus, western blotting to detect the CCNA2 and CDK2 levels in MG-63 following dimers treatment was further performed. The results showed that the levels of CDK2 and CCNA2 in MG-63 were dose-dependently reduced by Comp1 and Comp2, supporting the inhibitory effect of dimers on CDK2 and CCNA2 expression (**Figure 6G**).

**Figure 7:**
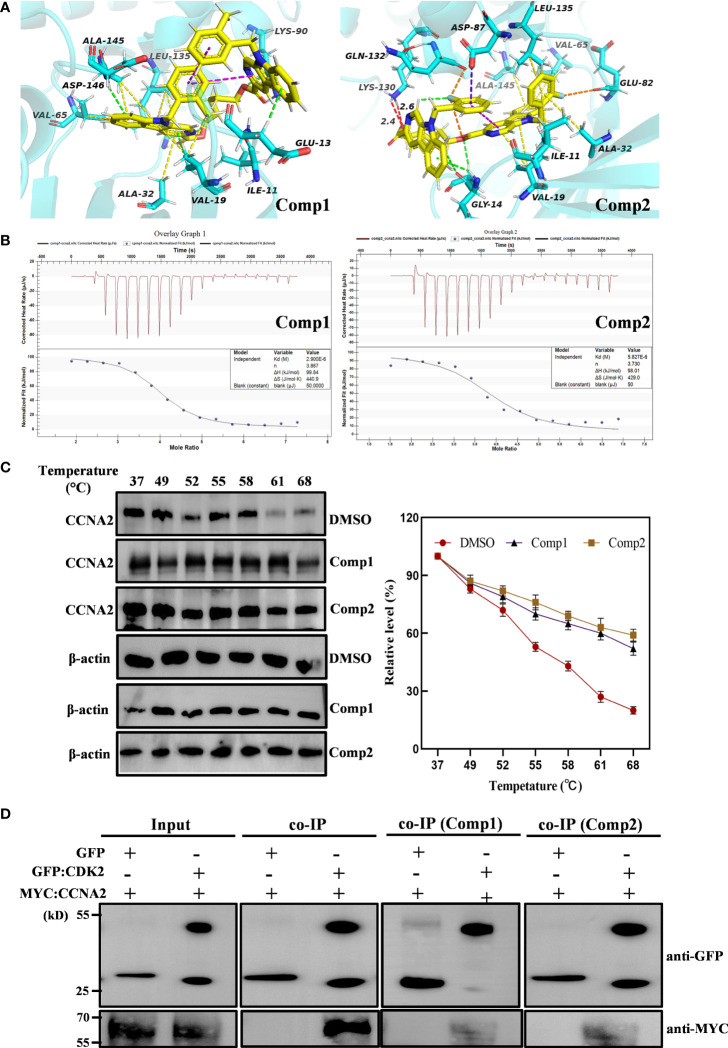
*β-*Carboline-3-carboxylic acid dimers directly inserted into CCNA2. (PDB ID: 1E9H). **(A)** CCNA2 backbone-ligand Comp1 interaction (color code for ligand: Yellow-C: White-H; Red-O; Blue-N); CCNA2 backbone-ligand Comp2 interactions (color code for ligand: Yellow-C; White-H; Red-O; Blue-N; cyans-F). **(B)** Isothermal titration calorimetry (ITC) enthalpograms of Comp1 and Comp2 binding to CCNA2. Titration data are presented as colored circles and fit as black solid lines. The molar ratio (N), equilibrium dissociation constant (KD), and enthalpy change (∆H) are shown in the inset. **(C)** The cell lysates were heated at the indicated temperature and protein was measured by Western blot. **(D)** Co-immunoprecipitation (co-IP) assay shows that Comp1 and Comp2 block the interaction of CCNA2 with CDK2. The interaction affinity between CCNA2 and CDK2 is significantly decreased by the presence of *β*-Carboline-3-carboxylic acid dimers.

Subsequently, the pharmacology network showed a direct interaction between dimers and CCNA2 was noted. Thus, further molecular docking analysis was performed on Comp1/Comp2 and CCNA2 (PDB ID: CCNA2: 1E9H; **Figure 7A**) to investigate their interaction relationship. The docking results showed that Comp1 could intercalate into the hydrophobic pocket of CCNA2 by hydrophobic and electrostatic interactions, with a high score of -9.2 kcal/mol. This hydrophobic pocket is the binding site for many inhibitors of this protein (**Figure 7A**). Observably, one of the carboline rings with N-9 substituents intercalated into the pocket and formed strong hydrophobic interactions with the Ala145, Leu135, Val65, and Ala32 residues of CCNA2, while another carboline ring interacted with Glu13 residues by weak electrostatic interactions (**Figure 7A**). Compared with Comp2, the binding pattern of Comp1 and CCNA2 was altered, the electrostatic interaction was enhanced, and the hydrophobic interaction was weakened. Certainly, Comp2 also interacted with CCNA2, with a credible score of -9.2 kcal/mol (**Figure 7A**).

**Figure 8:**
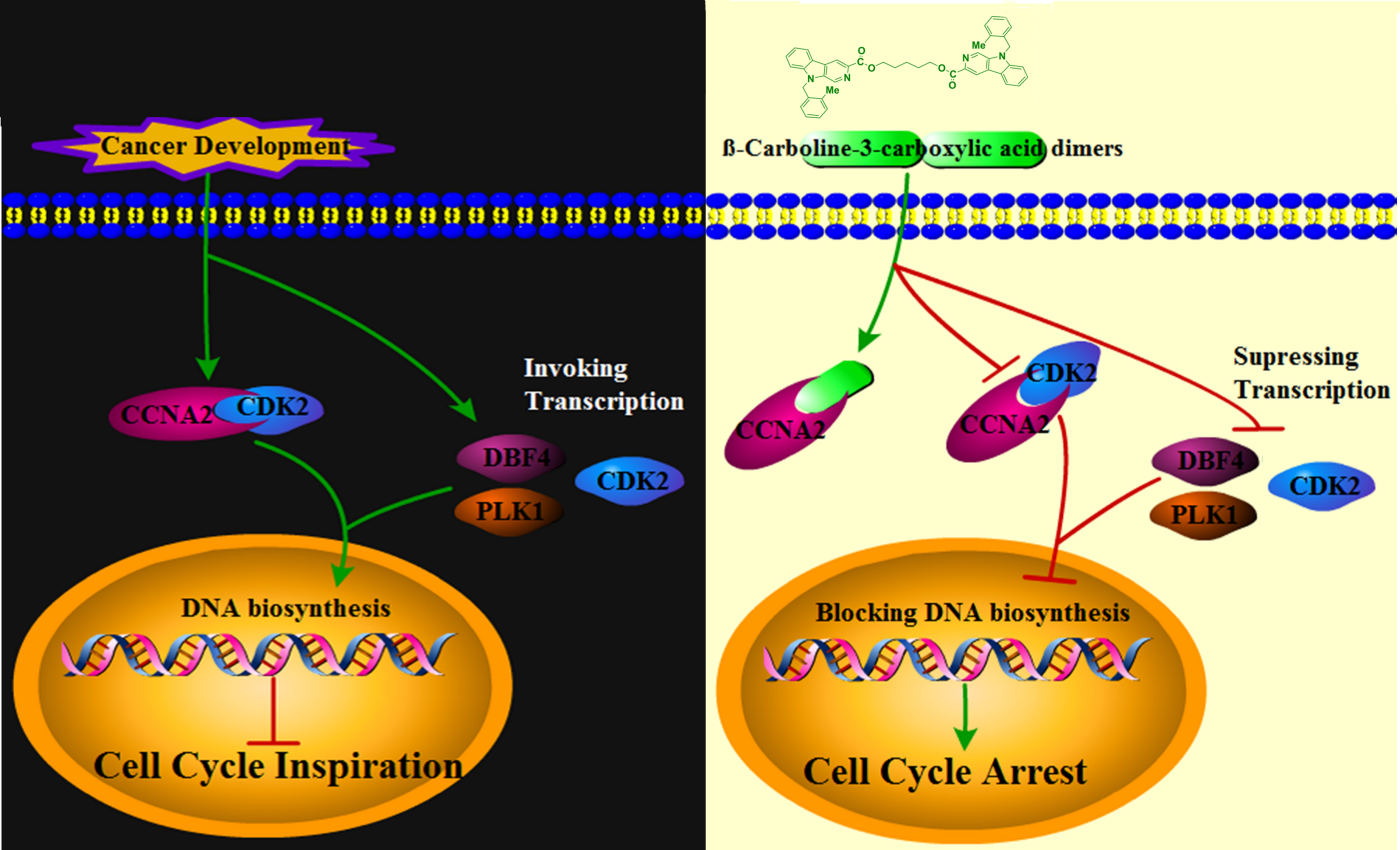
Schematic machinery of *β-*Carboline-3-carboxylic acid dimers inhibiting MG-63 cells.

To further validate the direct interaction between the selected *β*-carboline-3-carboxylic acid dimers and CCNA2, CETSA was performed. As expected, Comp1 and Comp2 also increased the thermal stability of the CCNA2 (**Figure 7C**). Subsequently, the CCNA2 protein was purified through a prokaryotic expression system. Furthermore, the interaction relationship between CCNA2 and *β-*carboline-3-carboxylic acid dimers using the ITC system was investigated. The results showed that *β-*carboline-3-carboxylic acid dimers directly interacted with the CCNA2 protein. Remarkably, *β-*carboline-3-carboxylic acid dimers also showed high-affinity binding events to CCNA2; Comp1 and Comp2 had dissociation constants of *K*_d1_ = 2.90 × 10^6^ nM and *K*_d2_ = 5.821 × 110^6^ nM, respectively (**Figure 7B**). It can be seen that CCNA2 and dimers interactions are caused by entropy-driven adsorption processes with endothermic processes (ΔH>0 & ΔS>0), a loss of enthalpy corresponding to a general hydrophobic force. Importantly, ITC and docking results reached a consensus that *β-*carboline-3-carboxylic acid dimers directly intercalate into the hydrophobic pocket of CCNA2 through hydrophobic interactions. These results supported our hypothesis that our dimers could target CCNA2 to inhibit SARC by affecting tumor immunity, proliferation, and apoptosis in MG-63 cells.CCNA2 could interact with CDK2 to initiate the cell cycle *in vivo* (38). Then, the detailed effect mechanisms of Comp1 and Comp2 on the interaction between CCNA2 and CDK2 were further investigated. The CCNA2 mature protein fused to MYC and CDK2 fused to GFP were co-expressed *in vivo*, and the interaction between them was confirmed using CoIP assay. Western blotting with anti-MYC and anti-GFP antibodies detected both CCNA2 and CDK2 in total proteins and proteins eluted from GFP beads (**Figure 7D**), supporting the interaction between CCNA2 and CDK2. However, the results further showed that this interaction affinity between CCNA2 and CDK2 was significantly decreased *in vivo* by adding Comp1 and Comp2 (**Figure 7D**). Thus, these results suggested that Comp1 and Comp2 could block the CCNA2-CDK2 interaction to cause arrest of cell cycle in SARC (**Figure 8**).

## Discussion

The sarcoma affects all ages, and the main treatment for SARC is still surgical excision and conventional chemotherapy for palliation (39). However, the drug resistance of patients and the lack of effective therapeutic targets dramatically restrict SARC therapy (14). Therefore, developing new cytotoxic agents for tumors and novel therapeutic strategies are urgent. Natural carbolines have inhibitory activity on cell proliferation against some carcinoma cells; thus, they can be used as precursors for developing new antitumor drugs. In our present study, the detailed antitumor mechanisms of *β-*carboline-3-carboxylic acid dimers were elucidated. The results showed that abnormal expression of genes functioned in apoptosis and cell cycle involved in the pathogenesis of SARC. Meanwhile, *β-*carboline-3-carboxylic acid dimers could inhibit the proliferation of MG-63 cells through promoting apoptosis and inducing arrest of cell cycle at S phase. Numerous genes that are involved in cell cycle were inhibited to downregulate in SARC by Comp1 and Comp2 treatments, especially *CDK2* and *CCNA2*. Both *CDK2* and *CCNA2* are involved in tumor immunity and are significantly associated with disease-free survival in SARC. Remarkably, the results showed that *β-*carboline-3-carboxylic acid dimers could directly interact with *CCNA2* through intercalation into its hydrophobic pocket to inhibit its expression and function. Moreover, Comp1 and Comp2 significantly blocked the interaction between CCNA2 and CDK2, leading to irreversible arrest of the cell cycle of cancer cells. Additionally, it was also identified that numerous transcripts responsible for regulating cell cycle including lncRNAs, miRNAs, and genes relevant to hedgehog and Ras signaling pathways involved in the complex network of SARC pathogenesis. Moreover, harmine, a typical carboline, also significantly affected their expression *in vivo*, implying the conserved anti-tumor mechanisms of *β-*carboline derivatives. Overall, this study contributes to our understanding of the antitumor mechanisms of carboline-like compounds and provides novel therapeutic targets and drug precursors for treating cancers.

### Antitumor mechanisms of the *β*-carboline dimers involved in the tumor cell cycle

Cell cycle irregularity is a common characteristic of human cancer, and its involvement in tumor immunity has been documented in various cancer types (40). Similarly, the cell cycle arresting capacity of some *β-*carboline derivatives through affecting PLK and CDK has been fully elucidated (41). Similarly, our flow cytometric cell cycle results for MG-63 also supported that *β-*carboline-3-carboxylic acid dimers have stronger cell cycle arresting activity. It has been proved that the activation of cyclin-CDK complexes is indispensable in driving cell cycle progression and tumor immunity (42). Increasing evidence suggests that CDK2 fundamentally functions in the cell cycle, DNA replication, and DNA damage of particular cancer types (43). Interestingly, the inhibition of CDK2 caused by carboline-like inhibitor 3-substituted 6-aminosulfonyl-*β-*carbolines results in the arrest of the cell cycle of MCF-7 cells (44). Further detailed characterization showed that harmine specifically inhibited CDK1, CDK2, and CDK5 in an ATP-competitive manner (45). Here, *β-*carboline-3-carboxylic acid dimers also inhibited the expression of *CDK2* and *PLK* which are involved in tumor immunity and pathogenesis, showing the broad-spectrum mechanism of carboline in arresting the cell cycle of tumor cells. Remarkably, our present study found that SARC-related cyclin CCNA2 is a new target of *β-*carboline-3-carboxylic acid dimers and both dimers could not only directly intercalate into CCNA2 but also inhibit its expression at both transcription and protein level. CCNA2 could activate CDK2 during the S phase, allowing the cell cycle to proceed normally, and it also could be recognized by high-avidity T-cells to perform function in tumor immunity (46). It was also found a significant relationship between *CCNA2* expression and CD4+ T cells in SARC. Considering the involvement of *CCNA2* in tumor immunity of various cancer cases, thus the dysregulation of *CCNA2* will result in disorders of the cell cycle and eventually promote the pathogenesis of tumors. Of note, the intercalation of *β-*carboline-3-carboxylic acid dimers into CCNA2 irreversibly changes the conformation and function of CCNA2, blocking the interaction between CCNA2 and CDK2 and disrupting the normal process of the cell cycle. Thus, the results showed that *β-*carboline-3-carboxylic acid dimers could dual-regulate the function of CCNA2, leading to the arrest of the cell cycle of MG-63 cells in the S phase.

Subsequently, docking results showed that Comp1 and Comp2 could interact with protein DBF4 in a similar pattern in which both Comp1 and Comp2 intercalate into the hydrophobic pocket of DBF4 through hydrophobic interactions, electrostatic interactions, and van der Waals forces (**Figure S4**). Six targets in disease-free survival of SARC could directly or indirectly interact with the *DBF4* which also significantly negatively correlated with CD4+ T cells. Remarkably, the docking evaluation score of Comp2-DBF4 was -9.7 kcal/mol, which was higher than that of Comp1-DBF4, which had a score of -9.1 kcal/mol. Importantly, the carboline ring near the outside of the pocket forms a weak hydrogen bond (2.3 Å) with the LYS139 residue in addition to the π-cation stacking interaction, which contributed to the binding interaction of Comp2 and DBF4 (**Figures S4A, B**). Although the real interaction between dimers and DBF4 needs further research, our research provided theoretical basis for identifying targets of carboline in treating cancer. Overall, these results displayed a credible interaction relationship between compound Comp1/Comp2 and CCNA2 protein, supporting our hypothesis that *β-*carboline-3-carboxylic acid dimers could directly interact with CCNA2 associated with the cell cycle and tumor immunity to inhibit the pathogenesis of SARC.

### Multilayer variations involved in carboline antitumor mechanisms

Currently, numerous studies suggested that the progression and severity of tumors are not caused by individual genetic aberration or deregulation of single signaling pathway but require the cooperation of various signaling pathways in cancer cells (47). It is feasible that simultaneous targeting of several oncogenic signaling pathways could provide us with more efficacious cancer treatments. TCGA, GEO, and targets of *β-*carboline-3-carboxylic acid dimer were integrated and showed that *β-*carboline treatment could cause alterations in the expression of genes involved in the hedgehog signaling pathway, Ras signaling pathway, and lysosomes, which are involved in tumor immunity (**Figure S2D**).

Metabolic reprogramming and its functional effects are well known for cancer cells (48). Tumor cells exploit or regulate gluconeogenesis enzymes, and the gluconeogenesis pathway is usually inhibited in cancers because it antagonizes glycolysis (48). Here, it was also found that exogenous application of harmine reprogrammed the glycolysis/gluconeogenesis pathway by affecting the expression of related genes. Among these genes, phosphoenolpyruvate carboxykinase 1 (*PCK1*) is the hub gene that is shared by the profiles of GEO, TCGA, and *β-*carboline-3-carboxylic acid dimer targets. PCK1 encodes an imperative enzyme involved in the glycolysis/gluconeogenesis pathway and tumor immunity, and PCK1 catalyzes the first rate-limiting reaction of gluconeogenesis in the cytoplasm (49). The various credible evidence that the expression abundance of *PCK1* was lower in SARC than that in normal cells. Interestingly, harmine and *β-*carboline-3-carboxylic acid dimer treatment also significantly affected *PCK1 in vivo* (**Figure S2D**). Additionally, harmine also inhibits nutrient-related metabolic pathways, including arginine and proline metabolism, steroid biosynthesis, and glycerophospholipid metabolism. Thus, harmine may inhibit tumors by suppressing the metabolisms relevant to nutrients, leading to a hostile environment for the tumor cell proliferation. However, the molecular mechanisms that underlie the metabolic reprogramming of cancer cells are complex (50), and further experiments are needed to validate the function of carbolines in tumor metabolism.

## Conclusions

The present results highlight that *β-*carboline-3-carboxylic acid dimers could intercalate into the hydrophobic pocket of CCNA2 directly and further competitively disrupt the interaction between CDK2 and CCNA2, which suppresses the transmission of cell cycle-related signals, leading to the profound arrest of the cell cycle in cancer cells. The results suggest that regulation of cyclin A2/CDK2 mRNA and protein levels may represent one important mechanism by which *β-*carboline-3-carboxylic acid dimers exert anticancer effects.

## Data availability statement

Publicly available datasets were analyzed in this study. This data can be found here: GSE150563 and The Cancer Genome Atlas.

## Author contributions

HM designed the experiments. HM, HY and ZL are involved in new experiments required by peer review. HM, YD, HY, ZC, and ZL drafted the original manuscript and prepared the table and figure. JD, DZ, YX, NL, and JW provided critical feedback. All authors contributed to the article and approved the submitted version.

## Funding

This work was supported by the National Natural Science Foundation of China (Grant No. 81773603).

## Acknowledgments

This study used the databases from TCGA Program and GEO, and the authors acknowledge the efforts of the corresponding institutes. The interpretation and reporting of these data are the sole responsibility of the authors. The financial support from the National Natural Science Foundation of China was gratefully acknowledged.

## Conflict of interest

The authors declare that the research was conducted in the absence of any commercial or financial relationships that could be construed as a potential conflict of interest.

## Publisher’s note

All claims expressed in this article are solely those of the authors and do not necessarily represent those of their affiliated organizations, or those of the publisher, the editors and the reviewers. Any product that may be evaluated in this article, or claim that may be made by its manufacturer, is not guaranteed or endorsed by the publisher.

## Abbreviations

SARC, sarcoma; CCNA2, Cyclin-A2; GSEA, gene set enrichment analysis; TCGA, The Cancer Genome Atlas; CDK2, Cyclin-dependent kinase 2; GEO, Gene Expression Omnibus; DEGs, Differentially expressed genes; DBF4, Protein DBF4; PLK1, Serine/threonine-protein kinase; MMP7, Matrilysin; TK1, Thymidine kinase; CLDN4, Claudin-4; SLC44A3, Choline transporter-like protein 3; PRKACB, cAMP-dependent protein kinase catalytic subunit beta; PCK1, Phosphoenolpyruvate carboxykinase.
