# Supplementary material for: β-Carboline dimers inhibit the tumor proliferation by the cell cycle arrest of sarcoma through intercalating to Cyclin-A2
Source: Front Immunol. 2022 Oct 17;13:922183. doi: 10.3389/fimmu.2022.922183 (PMC9618858; doi:10.3389/fimmu.2022.922183)
Supplement: Supplementary file 1 [file DataSheet_1.docx]

**Supplementary materials**

Supplementary Table 1 Primer sequences for RT-qPCR

| **Gene** | **Sequence (5′–3′)** |
| --- | --- |
| *CCNA2* | Forward: TTTTGACTTAGCTGCTCCAA  Reverse: AGGTATGGGTCAGCATCTAT |
| *DBF4* | Forward: AGGACATTAAGGATCTGGGA  Reverse: AGAAATTCGACCCAAGGTTT |
| *PLK1* | Forward: TGCTCTTCAATGACTCAACA  Reverse: TTCATCAAGGAGTTGGGATG |
| *CDK2* | Forward: CTTTGCTGAGATGGTGACTC  Reverse: GGCTTGTAATCAGGCATAGA |
| *MMP7* | Forward: CTTTGCTGAGATGGTGACTC  Reverse: GGCTTGTAATCAGGCATAGA |
| *TK1* | Forward: AAGCCATTTGGGGCCATCCT  Reverse: AATCACCTCGACCTCCTTCTCTG |
| *ERBB4* | Forward: TGTTATGCAGACACCATTCA  Reverse: CGTCCACATCCTGAACTAC |
| *PCK1* | Forward: TCGAAAGCAAGACGGTTATC  Reverse: AAATCCTCCTCTGACATCCA |
| *SLC44A3* | Forward: GCAAAACGCACTGAAAGAA  Reverse: TGAGATGGAGCAGGTATTTG |
| *CLDN4* | Forward: AAGACTTCTACAATCCGCTG  Reverse: GGAGTAAGGCTTGTCTGTG |
| *SORD* | Forward: GCATTCTGTTGGAATCTGTG  Reverse: ATCCCACTTTTTCGACTGTT |
| *PYGL* | Forward: CTCTGGAATTTTACATGGGC  Reverse: ATCCAATCCAAGCTGGTAAA |
| *PRKACB* | Forward: ATGGGATTGTCACGCAAAT  Reverse: TTCAAGTCCGGCATTATTCT |
| *DCK* | Forward: TACGGGGAAGAAATGAAGAG  Reverse: TCCAGTGTTAAGATAGGCAC |
| *CES1* | Forward: TACGGGGAAGAAATGAAGAG  Reverse: TCCAGTGTTAAGATAGGCAC |
| *UCK2* | Forward: TAGCTTCTACCGTGTCCTTA  Reverse: ATCTGGACTGTTTTCCCTTC |
| *FBP1* | Forward: CTAACAAGAAGAGCCCCAAT  Reverse: TGAATGTCTGTGGGAATGAC |
| *GLI3* | Forward: GCACCACTTCTAATGAGGAT  Reverse: GAAGGTTCCTCACTGACTTT |
| *STK4* | Forward: GACGGTACAGCTGAGGAA  Reverse: ACTTCTTCTGGTTGTTTGGT |
| *FGF9* | Forward: AAGTTGGGAACTATTTCGGT  Reverse: GGTGGTCACTTAACAAAACC |
| *PRKACA* | Forward: GCAGGAGAGCGTGAAAG  Reverse: CGTTCAAACTGATCCAAGTG |


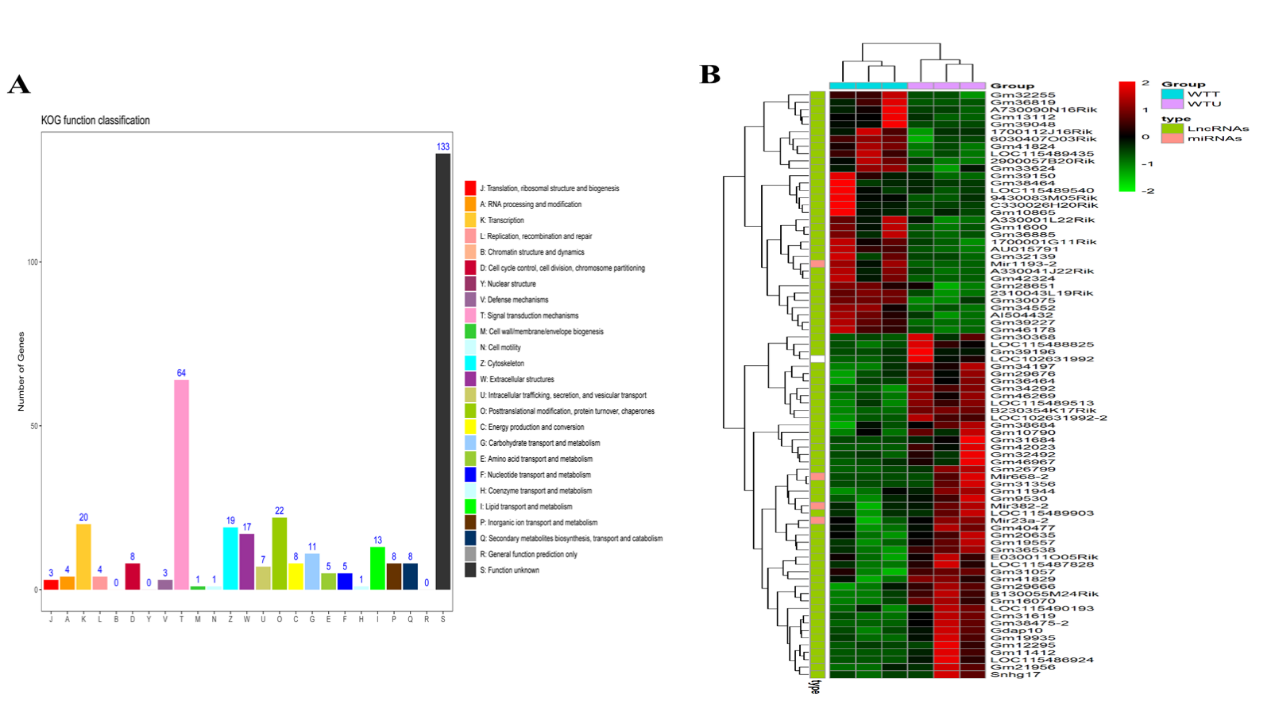


**Supplementary figure 1**(A) KOG enrichment analysis on differentially expressed genes, each column represented a single function enriched-terms in KOG analysis. (B) Expression abundance of LncRNAs and miRNAs under harmine treatment. Hot spots ranged from red (high expression) to green (low expression).


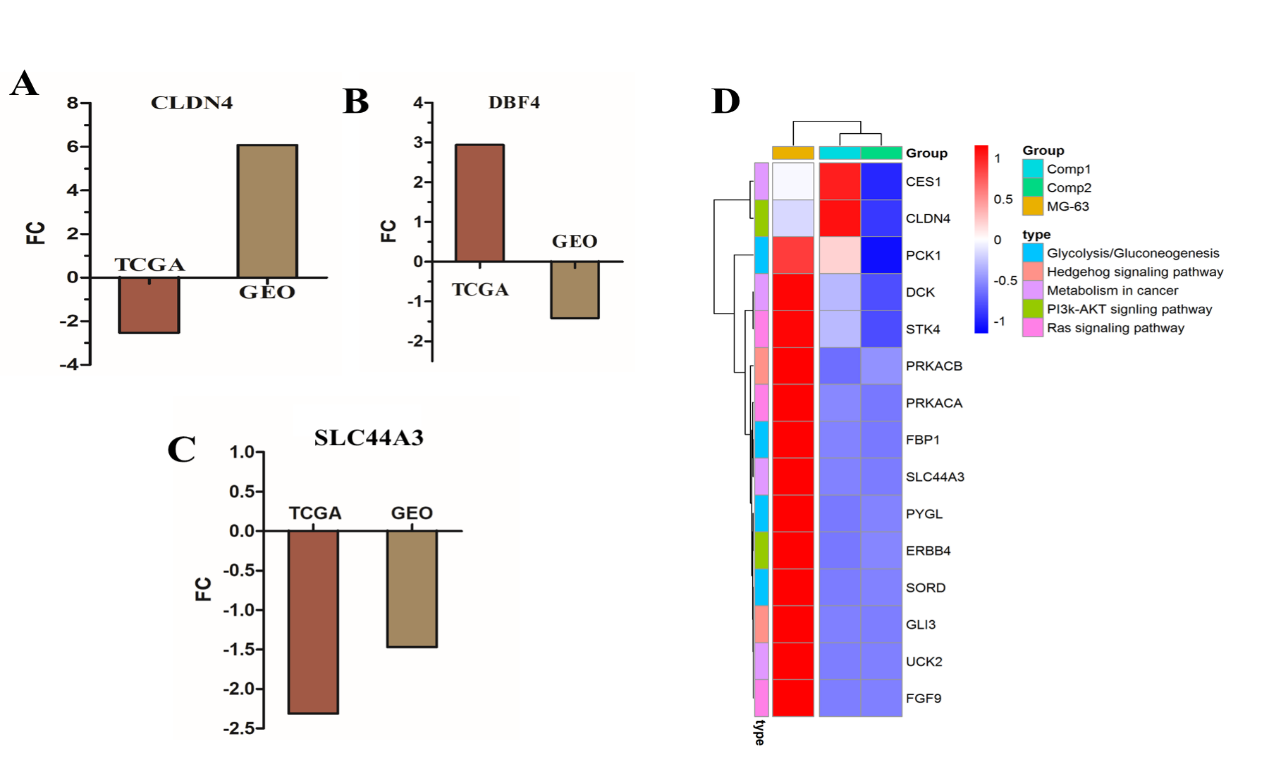


**Supplementary figure 2** (A-C) SLC44A3, DBF4, and CLDN4 expression level in TCGA and GEO.(D) Expression abundance of 15 important genes under β-Carboline-3-carboxylic acid dimers treatment. Hot spots ranged from red (high expression) to blue (low expression)


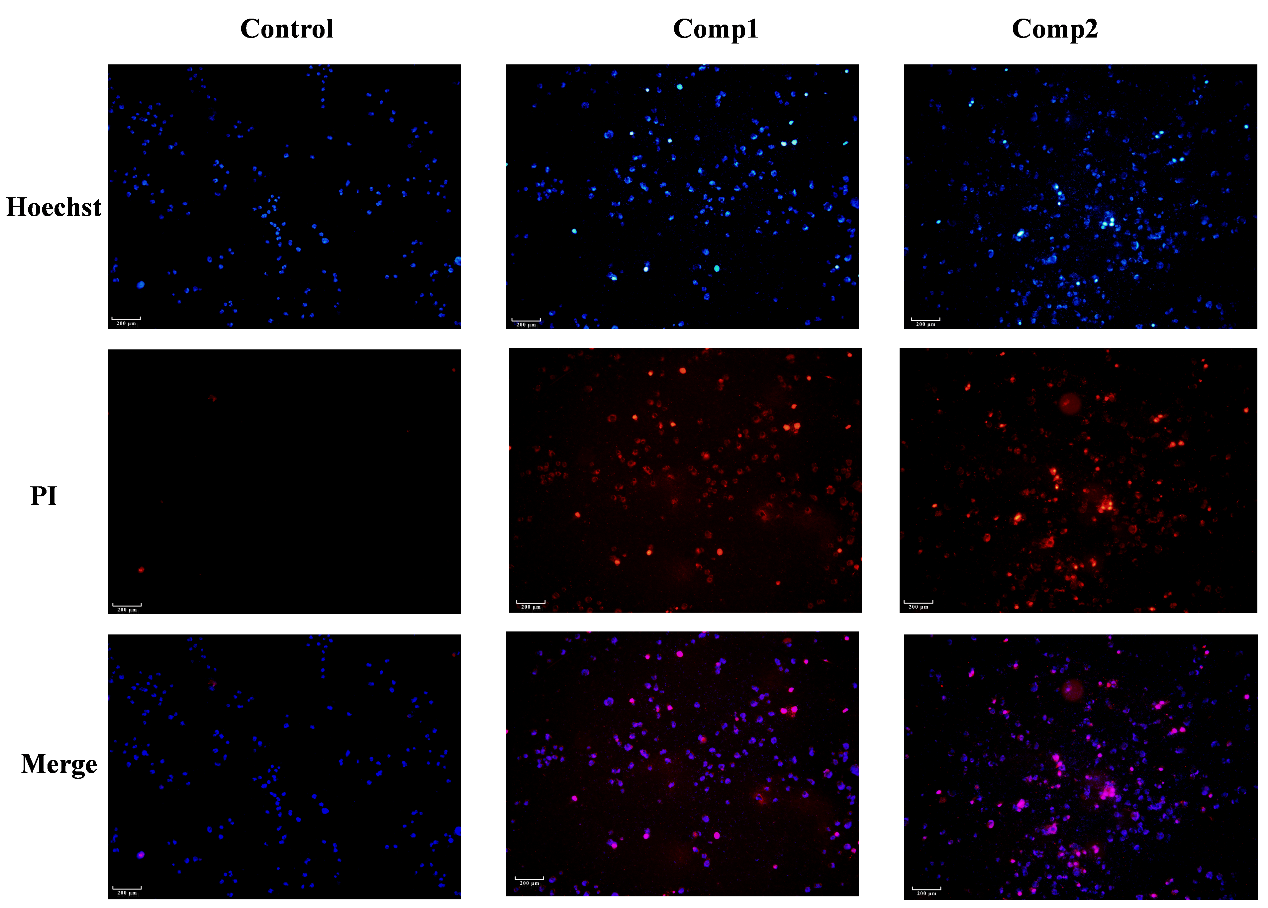


**Supplementary figure 3** Morphological observation was performed by Hoechst 33342/propidium iodide (PI) dual staining.


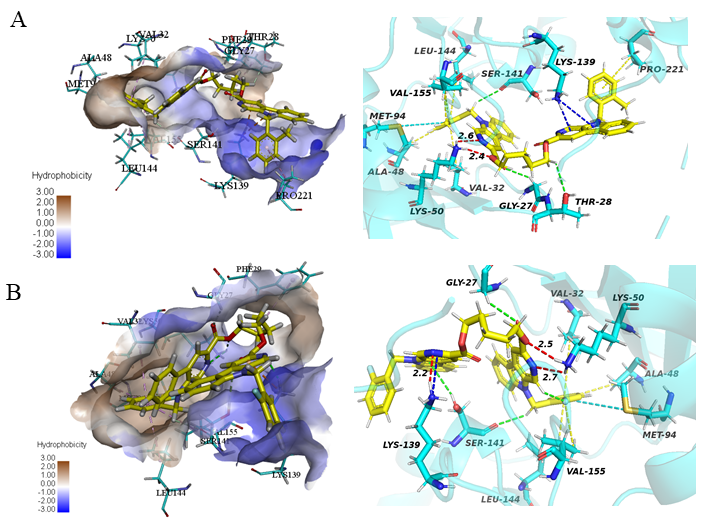


**Supplementary figure 4** **Schematic illustration for the binding mode between β-Carboline-3-carboxylic acid dimers and DBF4.** (A) DBF4 backbone-ligand comp 1 interactions (color code for ligand: Yellow-C; White-H; Red-O; Blue-N). (B) DBF4 backbone-ligand comp 2 interactions (color code for ligand: Yellow-C; White-H; Red-O; Blue-N; cyans-F).


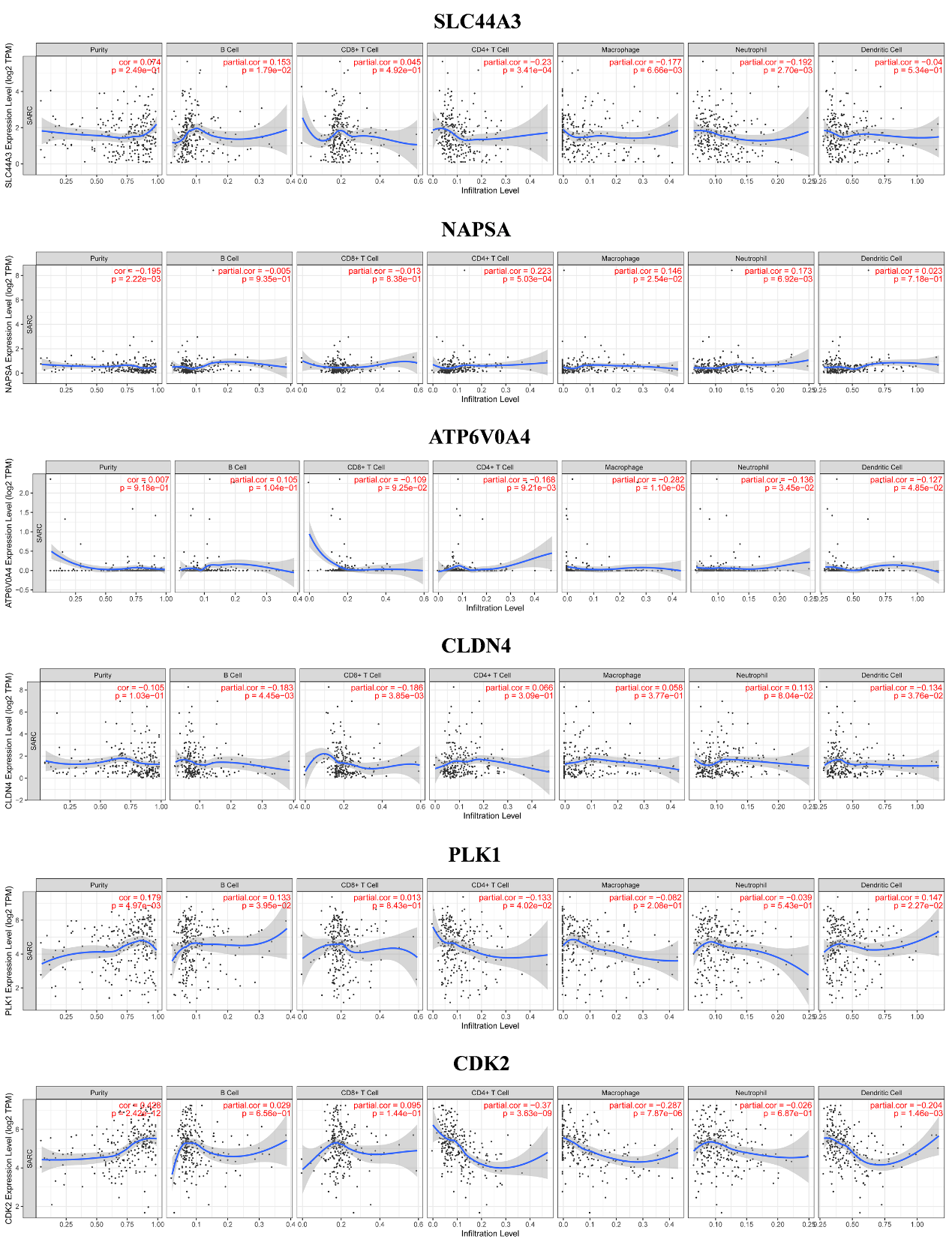


**Supplementary figure 5.** Correlation analysis between SARC-related genes and immune infiltration of 6 immune cell types. The potential correlation based on the TIMER algorithm between the expression level of SARC-related genes and the infiltration level of 6 immune cells was calculated using data from TCGA.


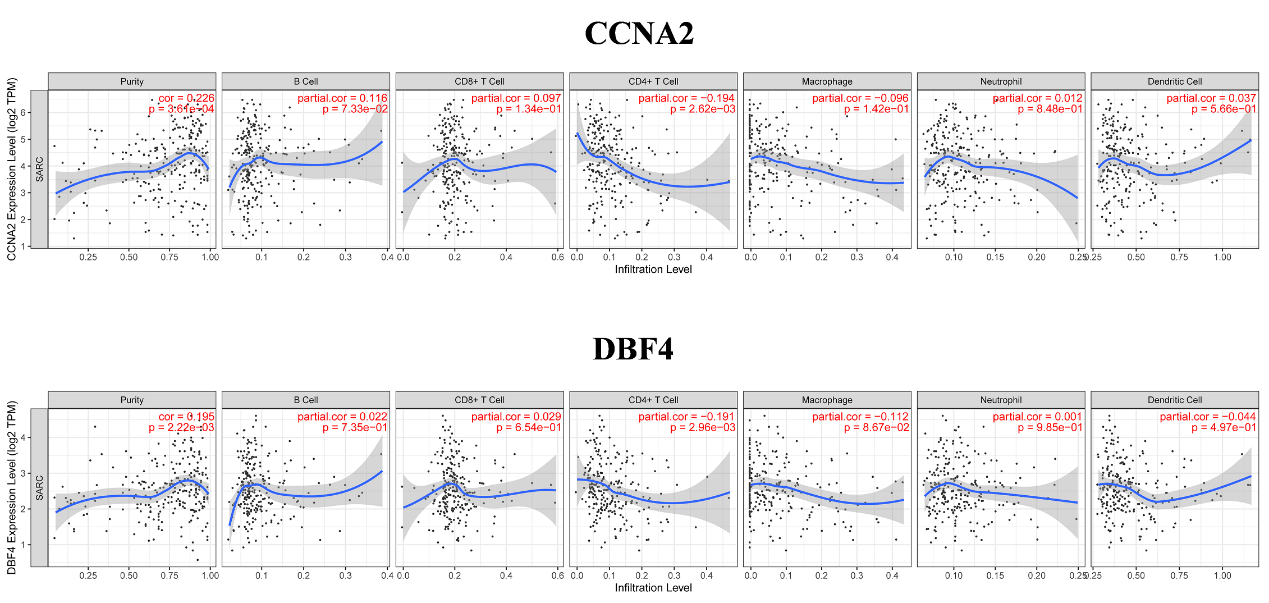


**Supplementary figure 6.** Correlation of CCNA2 and DBF4 expression and abundance of immune infiltrates in SARC.


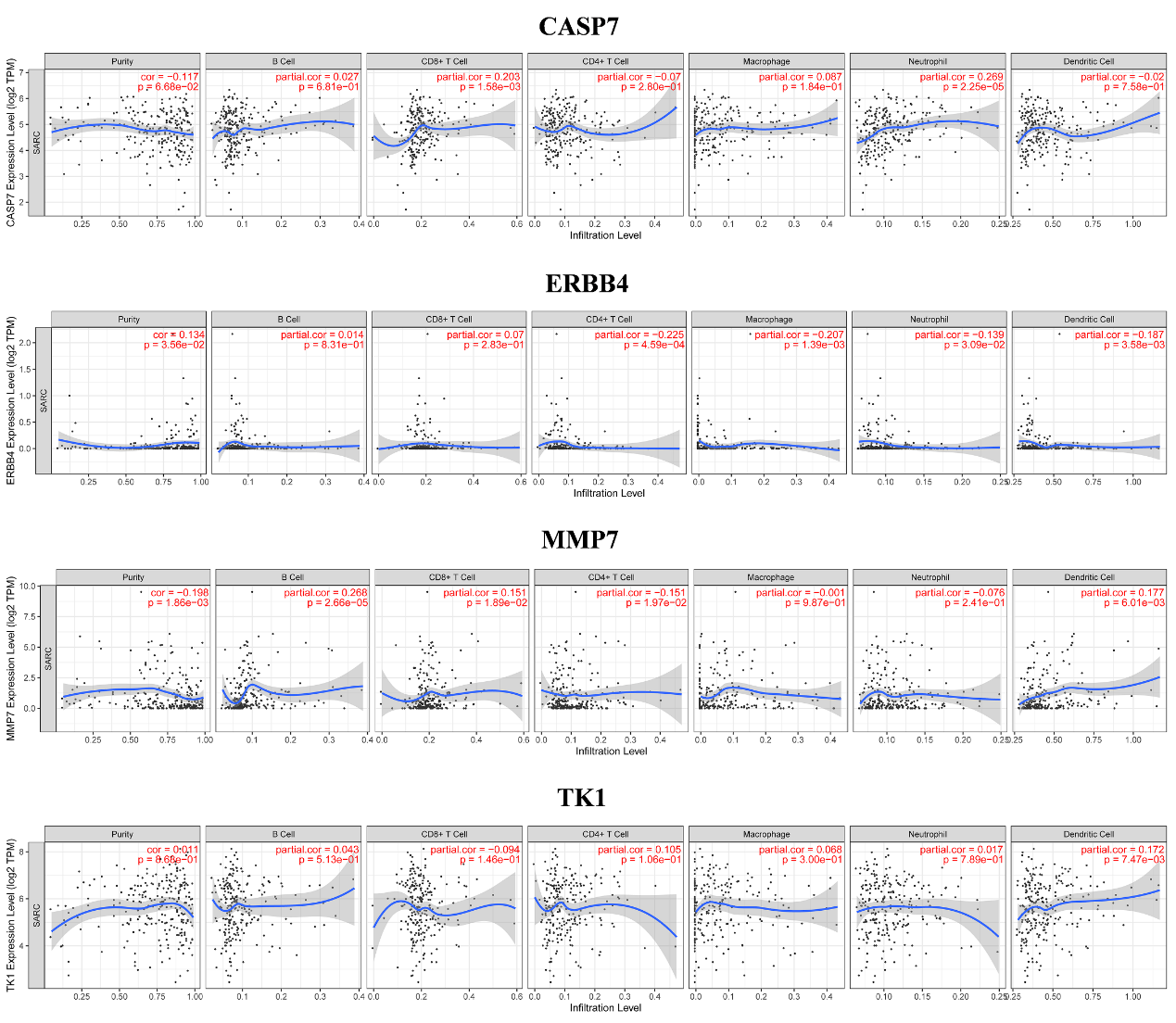


**Supplementary figure 7.** Correlation of hub-genes expression and abundance of immune infiltrates in SARC.
